# SOX30 specially prevents Wnt-signaling to suppress metastasis and improve prognosis of lung adenocarcinoma patients

**DOI:** 10.1186/s12931-018-0952-3

**Published:** 2018-12-04

**Authors:** Fei Han, Ming-qian Zhang, Wen-bin Liu, Lei Sun, Xiang-lin Hao, Li Yin, Xiao Jiang, Jia Cao, Jin-yi Liu

**Affiliations:** 1 Institute of Toxicology, College of Preventive Medicine, Third Military Medical University, 30 Gaotanyan Street, Shapingba District, Chongqing, 400038 People’s Republic of China; 2 Department of Emergency, Yan’an Hospital, Kunming Medical University, Kunming, Yunnan Province China

**Keywords:** Lung adenocarcinoma, Tumor-metastasis suppressor, Wnt-signaling, Prognostic biomarker, SOX30

## Abstract

**Background:**

Different histological subtypes of non-small cell lung cancer (NSCLC) show different molecular characteristics and responses to therapeutic strategy. Identification of specific gene, clarification of its special roles and molecular mechanisms are crucial for developing new therapeutic approach for particular subtype patients.

**Methods:**

Surgical specimens of 540 NSCLC patients were recruited. Immunohistochemistry was used to detect SOX30 expression, and correlations with clinical parameters were analyzed. Functional experiments and gene ontology analysis were performed to investigate roles of SOX30. Network analysis, TOP/FOP-Flash assays, luciferase reporter assays and ChIP-PCR assays were performed to determine the mechanism. Survival analyses were calculated by Kaplan-Meier and Cox regression. Recovery experiment was investigated the importance of the target of SOX30.

**Results:**

SOX30 expression is closely associated with histological types of NSCLC, and metastasis of adenocarcinoma (ADC) patients but not of squamous cell carcinoma (SCC) patients. SOX30 strongly inhibits cancer cell migration and invasion in ADC cell lines, whrereas not affects cell migration and invasion in SCC cell lines. The genes associated with SOX30 preferentially enrich in metastasis process and Wnt-signaling in only ADC patients. Consistently, SOX30 is negatively associated with the expression of Wnt-signaling and metastasis-related gene CTNNB1 (β-catenin) in ADC, but not in SCC. At the molecular level, SOX30 represses Wnt-signaling by directly transcriptional inhibition of CTNNB1 in ADC, and also not in SCC. In the clinical, SOX30 is a favorable and independent prognostic factor in ADC patients, whereas is an unfavorable and independent prognostic factor in SCC patients. Moreover, SOX30 expression is a double face early-stage prognostic biomarker in ADC and SCC patients. In addition, forcible restoration of CTNNB1 indeed can inhibit the anti-metastatic role of SOX30 in ADC patients.

**Conclusions:**

In early-stage ADC patients, elevated SOX30 expression inhibits tumor-metastasis by directly binding to CTNNB1 promoter resulting in a favorable prognosis of these patients. However, in early-stage SCC patients, SOX30 has no inhibitory role on tumor-metastasis due to not binding to CTNNB1 promoter leading to an unfavorable prognosis of the patients. This study highlights a special role and prognostic value of SOX30 in ADC, providing a novel therapeutic target for particular subtype NSCLC patients.

**Electronic supplementary material:**

The online version of this article (10.1186/s12931-018-0952-3) contains supplementary material, which is available to authorized users.

## Background

Lung cancer, as the most common malignancy, is the leading cause of cancer-associated mortality [1]. The prognosis of lung cancer patients is very poor and the five year survival rate is no more than 10% [2, 3]. The primary histological subtype is the non-small cell lung cancer (NSCLC) constituting over 80% of lung cancer [4]. As the two major subtypes of NSCLC, adenocarcinoma (ADC) and squamous cell carcinoma (SCC) have been considered to be similar in postsurgical prognosis and chemotherapeutic response for very long time. However, a number of recent studies have found that ADC and SCC have a variety of differences not only in postsurgical prognosis and chemotherapeutic response, but also in molecular characteristics and therapeutic strategies [5–9]. These studies demonstrate that efficacy of therapy options varies for different subtype patients, highlighting the importance of characterizing molecular abnormalities in different tumor-subtypes [10–14]. Thus, identification of novel gene and clarification of its specific function and clinical value are crucial for developing new and special therapeutic approach for particular subtype patients of NSCLC.

SOX30, as an important SOX (sex determining region Y-box) family factor, has been reported to be involved in spermatogenesis [15–18]. For many years, the roles of SOX30 on tumorigenesis are completely unknown. Recently, SOX30 has been identified as a key participant in tumorigenesis of several type tumors including colon cancer and lung cancer [19–23]. However, the value and precise role of SOX30 in different subtype patients of NSCLC remain largely unexplored.

In our present study, we assessed an unknown clinical significance, specific tumor suppressor role and molecular mechanism of SOX30 in different subtype NSCLC using clinical association analysis, prognostic analysis and differentially expressed cell models. Our findings demonstrate that SOX30 is a special tumor-metastasis suppressor and early-stage favorable prognostic factor by directly repressing Wnt/β-catenin (CTNNB1) signaling activity only in ADC patients, providing novel insights into and specifically potential molecular-targeted strategy for NSCLC therapy.

## Methods

### Cell lines

The human ADC cell lines: A549 and SPC-A-1 and SCC cell lines: H520 and H2170 were obtained from the Cell Bank of Chinese Academy of Science (CBCAS, Shanghai, China) and the American Type Culture Collection (ATCC, Manassas, VA, USA), cultured in F12K (Sigma, St. Louis, MO, USA) or PMI-1640 (HyClone, Logan, UT, USA) media supplemented with 10% fetal bovine serum (FBS) (Gibco, Carlsbad, CA, USA), and incubated at 37 °C in 5% CO_2_ cell incubator.

### Patient samples

A total of 540 NSCLC (275 ADC, 231 SCC and 34 large cell carcinoma [LCC]) patients undergone surgical resection between 2004 and 2014 were recruited from Daping Hospital or Southwest Hospital Affiliated to Third Military Medical University in Chongqing, China. The clinico-pathologic information was retrieved from the electronic medical records of patients, which includes age, gender, histological type, histological grade, clinical stage, tumor size, tumor location, lymph node, tumor diameter, tumor metastasis and Overall survival (OS). OS was defined as the period from surgery to death or the last observation. The clinico-pathologic parameters also include the expression of CTNNB1. The study was approved by the ethics committee of Daping Hospital and Southwest Hospital. The informed consent was signed by the patients participating in the study. All experiments were performed in accordance with the approved guidelines of the Third Military Medical University.

### Tissue microarray generation and immunohistochemical (IHC) analysis

The tissue microarray was generated and IHC analysis was performed as a previous study [20]. IHC staining was performed using the antibody against SOX30 (1:100, Santa Cruz Biotechnology, Germany), and was evaluated and defined as positive when immunoreactivity ≥5%. Positive percentage of cell staining was classified into 5 categories: < 5% positive cells as 0 score; 5 to 25% as 1 score; 26 to 50% as 2 score; 51 to 75% as 3 score and ≥ 76% as 4 score. Intensity of cell staining was graded as negative for 0 score, weak for 1 score, moderate for 2 score and strong for 3 score. SOX30 expression levels were calculated by product of category for the positive percentage and grade for the intensity of staining [24, 25]. The range of the calculation for SOX30 expression was therefore 0–12 score. The expression of CTNNB1 was measured by IHC staining using the antibody against CTNNB1 (1:150, Santa Cruz Biotechnology). IHC staining of CTNNB1 was then evaluated and defined as positive when immunoreactivity ≥5%, otherwise it was identified as negative. All biopsy tissues of IHC staining were independently reviewed by two blinded pathologists.

### RNA isolation and RT-PCR

Total RNA was extracted from A549, SPC-A-1, H520 and H2170 cells. Approximately 3.0 μg of RNA was treated with DNase I (Invitrogen Preservation, Carlsbad, CA, USA) to eliminate the genomic DNA, and then was reverse-transcribed to cDNA. RT-PCR (Reverse transcription polymerase chain reaction) was performed as previously described [19]. The following primers were used in this study. SOX30 forward primer (5′-3′): GAT GTC CCG CTC ACC GTG TTG C, SOX30 reverse primer (5′-3′): GAC AGG GCT TGG GCT CTG GAC T; β-actin forward primer (5′-3′): GAG CTA CGA GCT GCC TGA CGG, β-actin reverse primer (5′-3′): CCT AGA AGC ATT TGC GGT GG.

### Western blotting analysis

Western blotting (WB) analysis was performed as described previously [19]. The primary antibodies used this study were: SOX30 rabbit polyclonal antibody (1:800; Santa Cruz Biotechnology, sc-20,104), CTNNB1 mouse monoclonal antibody (1:800; Santa Cruz Biotechnology, sc-7963). Secondary antibodies were horseradish peroxidase (HRP)-conjugated (1:4000, Jackson, PA, USA).

### Plasmid construction and cell transfection

The plasmid construction and cell transfection were performed as described previously [19]. Briefly, the human SOX30 plasmid was constructed by synthesis, subcloned into pIRES2-EGFP vector (Invitrogen) and was validated by sequencing. The plasmids were transfected into A549, SPC-A-1, H520 and H2170 cells using ViaFect Transfection Reagent (Promega). The stably transfected cells were screened under G418 (Calbiochem, CA, USA), and the cell clones were obtained by cylinder method.

### Migration and invasion assays

The migration and invasion activities of cancer cells were evaluated using transwell assays in 24-well plates (8 μm, Corning, Acton, MA, USA) to assess the cell migration (without matrigel) and invasion (with matrigel). A549, SPC-A-1, H520 and H2170 cells stably transfected with pIRES2-EGFP-SOX30 or pIRES2-EGFP empty vector in serum-free medium were seeded into upper well of the chamber at 2 × 10^4^ cells/well. Lower well of the chamber contained fully media supplemented with 10% FBS. The migrated cells at the lower chamber were stained with 0.1% crystal violet, and counted under an inverted microscope after 12 h for migration or 18 h for invasion. All experiments were repeated thrice in triplicate for each.

### Gene ontology analysis

Gene ontology (GO) analyses were performed using DAVID 6.7 (https://david.ncifcrf.gov/) [26, 27]. The genes associated with SOX30 were selected for analysis when the *p*-value of correlation less than 0.05. The corresponding GO terms and pathways were enriched in these related genes.

### Network analysis

The PPINs, human protein-protein interaction networks, were first downloaded from the Human Protein Reference Database. The igraph package of statistical language R was used to analyze the functional profiling. The network of visualization and Cytoscape were applied to find the putative target genes. The Markov cluster algorithm was performed to identify the closely connected modules within the networks.

### TOP/FOP-flash assays and luciferase reporter assays

The ADC cell line (SPC-A-1) and SCC cell lines (H520 and H2170) were transfected with CTNNB1-promoter, pIRES2-EGFP empty vector, pIRES2-EGFP-SOX30 and TOP-Flash with wild-type TCF/LEF binding sites/FOP-Flash with mutated TCF/LEF binding sites plasmids which purchased from Addgene (12,456 and 12,457, Cambridge, MA, USA). Luciferase reporter assays were performed using the fluorescence microplate reader measurement system Varioskan LUX (Thermo Fisher, Waltham, MA, USA) and a Dual-luciferase reporter kit (Promega). The *Renilla reniformis* luciferase reporter was selected as an internal control. The luciferase activities of luciferase reporter assay were analyzed at 36 h after transfection. All the experiments were repeated thrice.

### Chromatin-immunoprecipitation RT-PCR assay

Chromatin-immunoprecipitation (ChIP) assay was performed according to the manufacturer’s protocol of the ChIP assay kit (Cell Signaling Technology). Briefly, 5 × 10^6^ SPC-A-1, H520 and H2170 transfected cells were fixed in 1% formaldehyde. The fixed cells were digested with micrococcal nuclease and chromatin immunoprecipitated after analysis of chromatin digestion. Then the samples were eluted of chromatin and purified DNA. The immunoprecipitated DNA and the input DNA were used as the templates for RT-PCR using the following primers. CTNNB1-p forward primer 2 (5′-3′): TCT TAA CCA ATT TCA AGA GTG CCT; CTNNB1-p reverse primer 2 (5′-3′): GGC GTT TTC AGG TAC TGA TTC C.

### Statistical analysis

Statistical analyses were performed using SPSS 13.0 software (SPSS, Inc., Chicago, IL, USA). The expression was categorized as high or low according to the median score. Survival analyses were calculated by Kaplan-Meier methods. Cox regression was used for multivariate analysis of prognostic predictors. The differences between two or three groups were analyzed using Chi-square test, Student’s t-test or One-way ANOVA. The *p*-values of less than 0.05 were considered statistically significant.

## Results

### SOX30 expression is highly associated with histological types of NSCLC

**Fig. 1:**
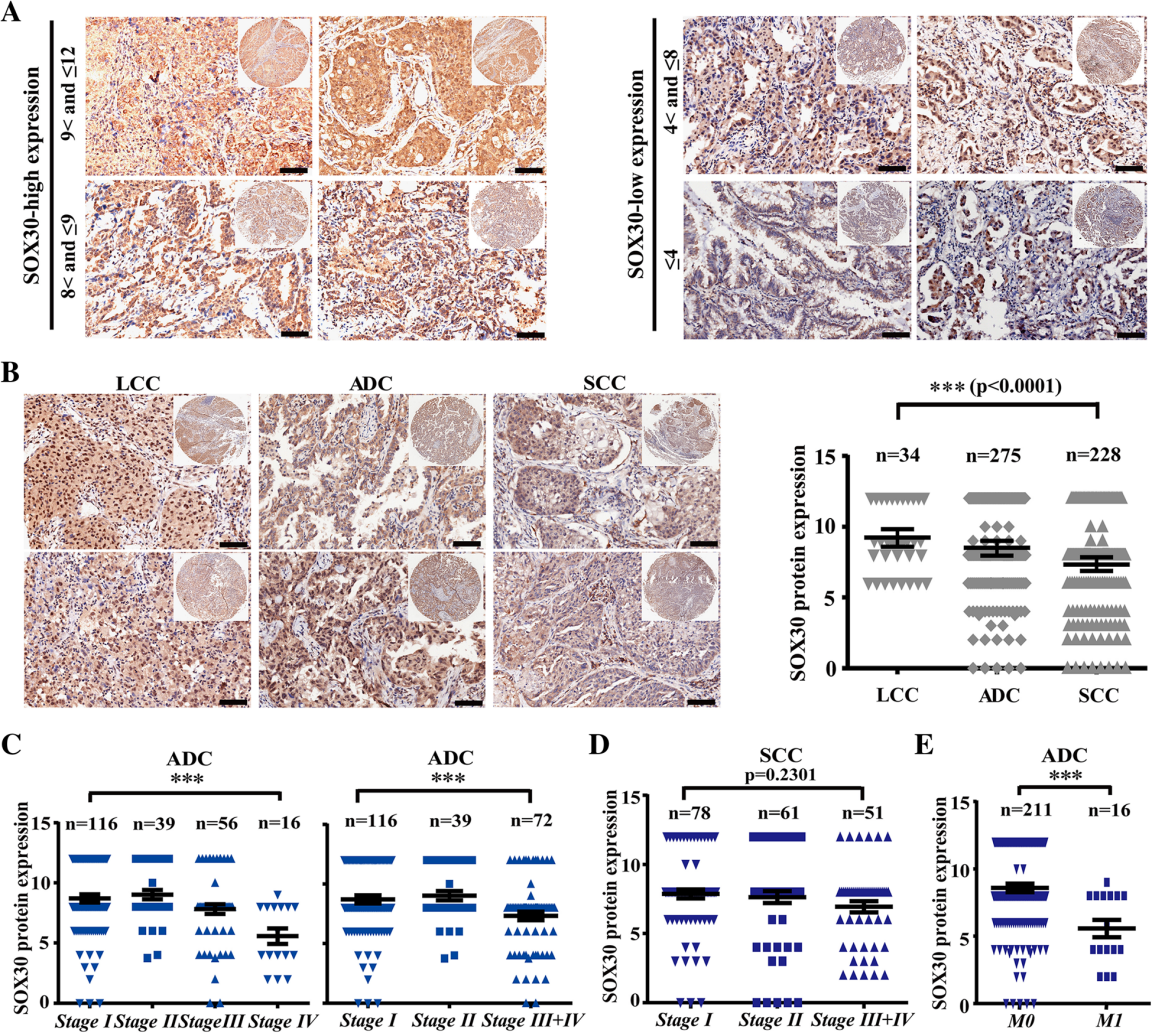
SOX30 expression is associated with histological types of NSCLC and metastasis of ADC*.* **a** SOX30 expression is presented at different score levels. SOX30 low expression group contains the ≤8 score patients. SOX30 high expression group contains the 8 < and ≤ 12 score patients. Scale bars represent 20 μm. **b** SOX30 expression was analyzed in LCC, ADC and SCC patients. The expression of SOX30 was significant reduced from LCC, ADC to SCC patients. Scale bars represent 20 μm. **c** SOX30 expression was determined in ADC patients at stage I, II, III and IV. The *p* value was measured with pearson chi-square tests and Student’s t-tests. *** represents *p* < 0.001. **d** SOX30 expression was measured in SCC patients at stage I, II, III and IV. The *p* value was measured with pearson chi-square tests and Student’s t-tests. **e** SOX30 expression was compared between non-metastatic and non-metastatic ADC patients. M0 represents non-metastatic cancers. M1 represents metastatic cancers

To determine the expression level of SOX30 in tumor tissues of different NSCLC histological types, we probed SOX30 expression using immunohistochemistry (IHC) in lung tumor tissues of 540 NSCLC patients including 275 ADC, 231 SCC and 34 LCC on tissue microarrays. Based on the quantification of positive percentage and intensity of staining, the 540 NSCLC patients were divided into two groups, the SOX30 high-expression group (scores 8 < and ≤ 12) and the SOX30 low-expression group (scores ≤8) (Fig. 1a). After analyzing SOX30 expression with clinicopathologic characteristics of these patients, we found that SOX30 was closely correlated with histological types of NSCLC (*p* = 0.000, Additional file 1: Table S1). The percentage of SOX30 high-expression samples was decreased from LCC patients (61.76%, 21/34), ADC patients (34.18%, 94/275) to SCC patients (18.86%, 43/228, three SCC samples were missing due to stripping) (Additional file 1: Table S1). The expression of SOX30 was also significant reduced from LCC (9.12 ± 2.27), ADC (8.52 ± 2.88) to SCC (7.39 ± 3.01) patients (Fig. 1b). These data demonstrate that SOX30 expression is associated with histological types of NSCLC.

### SOX30 is closely associated with clinical stage and metastasis of ADC patients not of SCC patients

As different expression of SOX30 in ADC and SCC patients, we further investigated the associations between SOX30 and clinicopathologic features in 275 ADC and 228 SCC patients, respectively. SOX30 expression was associated with clinical stage (*p* = 0.023), tumor size (*p* = 0.012) and long-distance metastasis (*p* = 0.022) in ADC patients (Table 1). Whereas, it was not associated with clinical stage (*p* = 0.315), tumor size (*p* = 0.060) and long-distance metastasis (*p* = 0.500), and only associated with histological grade (*p* = 0.001) in SCC patients (Additional file 1: Table S2). The percentage of SOX30 high-expression samples was significantly decreased from stageI (36.21%, 42/116), II (35.90, 14/39), III (23.21, 13/56) to IV (6.25%, 1/16) groups in ADC patients (*p* = 0.023) (Table 1), but was not statistically different among stage I (22.08%, 17/77), II (21.67%, 13/60) and III + IV (12.00%, 6/50) groups in SCC patients (*p* = 0.315) (Additional file 1: Table S2). Accordingly, SOX30 expression significantly decreased from stage I to IV groups in ADC patients (*p* = 0.0002), but was not statistically different among stage I, II and III + IV groups in SCC patients (Fig. 1c and d). Moreover, SOX30 expression was much lower in the ADC patients with metastasis than in the ADC patients without metastasis (Fig. 1e). These results suggest that SOX30 is associated with metastasis only in ADC patients.

**Table 1:** Correlation of SOX30 expression with clinicopathologic features in ADC patients (*n* = 275)

Clinical feature	Total	SOX30 expression		*P* value
		High (*n* = 94)	Low (*n* = 181)	
Age (years)				
≤ 60	151	56	95	0.328
> 60	121	38	83	
Clinical stage				
I	116	42	74	**0.023**
II	39	14	25	
III	56	13	43	
IV	16	1	15	
Tumor size				
T1–2	213	76	137	**0.012**
T3–4	31	4	27	
Lymph node status				
N0	128	45	83	0.160
N1–3	95	25	70	
Metastasis				
M0	230	78	152	**0.022**
M1	16	1	15	
Gender				
Male	152	52	100	0.991
Female	123	42	81	
Histological grade				
1	28	7	21	0.486
2	108	40	68	
3	70	25	45	
Tumor diameter				
≤ 4 cm	196	72	124	0.082
> 4 cm	71	18	53	
Location				
Left	125	46	79	0.426
Right	149	48	101	

The *p* values were measured with Pearson chi-square tests

The tumor clinical stage, tumor status, lymph node status and metastasis were classified according to the international system

All statistical tests are two sided

The entries in boldface indicate statistically significant

### The anti-metastatic role of SOX30 exists in ADC cell lines not in SCC cell lines

**Fig. 2:**
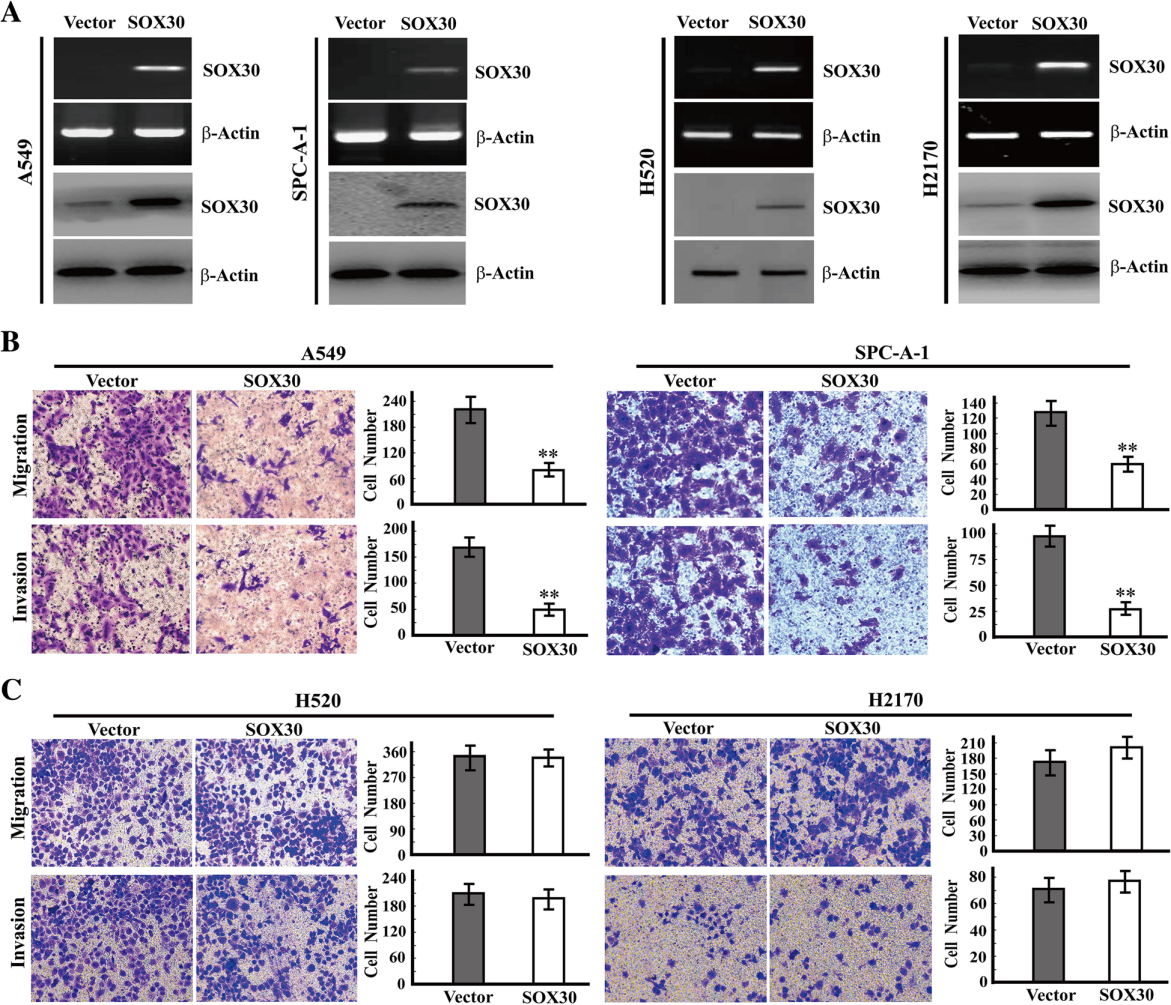
SOX30 inhibits cancer cell migration and invasion in ADC cell lines but not in SCC cell lines. **a** The ectopic expression of SOX30 in A549, SPC-A-1, A520 and H2170 cell lines by RT-PCR and WB. ACTIN represents β-actin. **b** Analyses of the migrated and invaded cells by transwell assays in A549 and SPC-A-1 cell lines. The cells in the lower chamber were fixed, stained and determined by the average count of five random microscopic fields. Error bars indicate SEM. The *p* value was measured with Student’s t-tests. ** represents *p* < 0.01. **c** Analyses of the migrated and invaded cells by transwell assays in A520 and H2170 cell lines. The cells in the lower chamber were fixed, stained and determined by the average count of five random microscopic fields. Error bars indicate SEM. ** represents *p* < 0.01. The p value was measured with Student’s t-tests

Different associations of SOX30 expression with metastasis in ADC and SCC patients suggested that SOX30 might play different roles on tumor metastasis in ADC and SCC. To investigate the role of SOX30 on cancer cell metastasis, we generated gain-of-function cell models in ADC cell lines (A549 and SPC-A-1) and SCC cell lines (H520 and H2170) by transfecting SOX30 or empty vector plasmids (Fig. 2a), and determined cell migration and invasion by transwell assays. The ability of cancer cell migration was strongly inhibited in SOX30-transfected cells compared with empty vector-transfected cells in A549, SPC-A-1 and LTEP-a-2 cell lines (Fig. 2b) [23]. The cancer cell invasion ability was also clearly reduced in SOX30-transfected A549, SPC-A-1 and LTEP-a-2 cells (Fig. 2b) [23]. However, there was no difference in cancer cell migration and invasion between SOX30-transfected and empty vector-transfected H520 and H2170 cells (Fig. 2c). These results demonstrate that the inhibitory role of SOX30 on metastasis seems to appear only in ADC cell lines, but not in SCC cell lines.

### The genes associated with SOX30 preferentially enrich in metastasis process and Wnt-signaling pathway in ADC patients

**Fig. 3:**
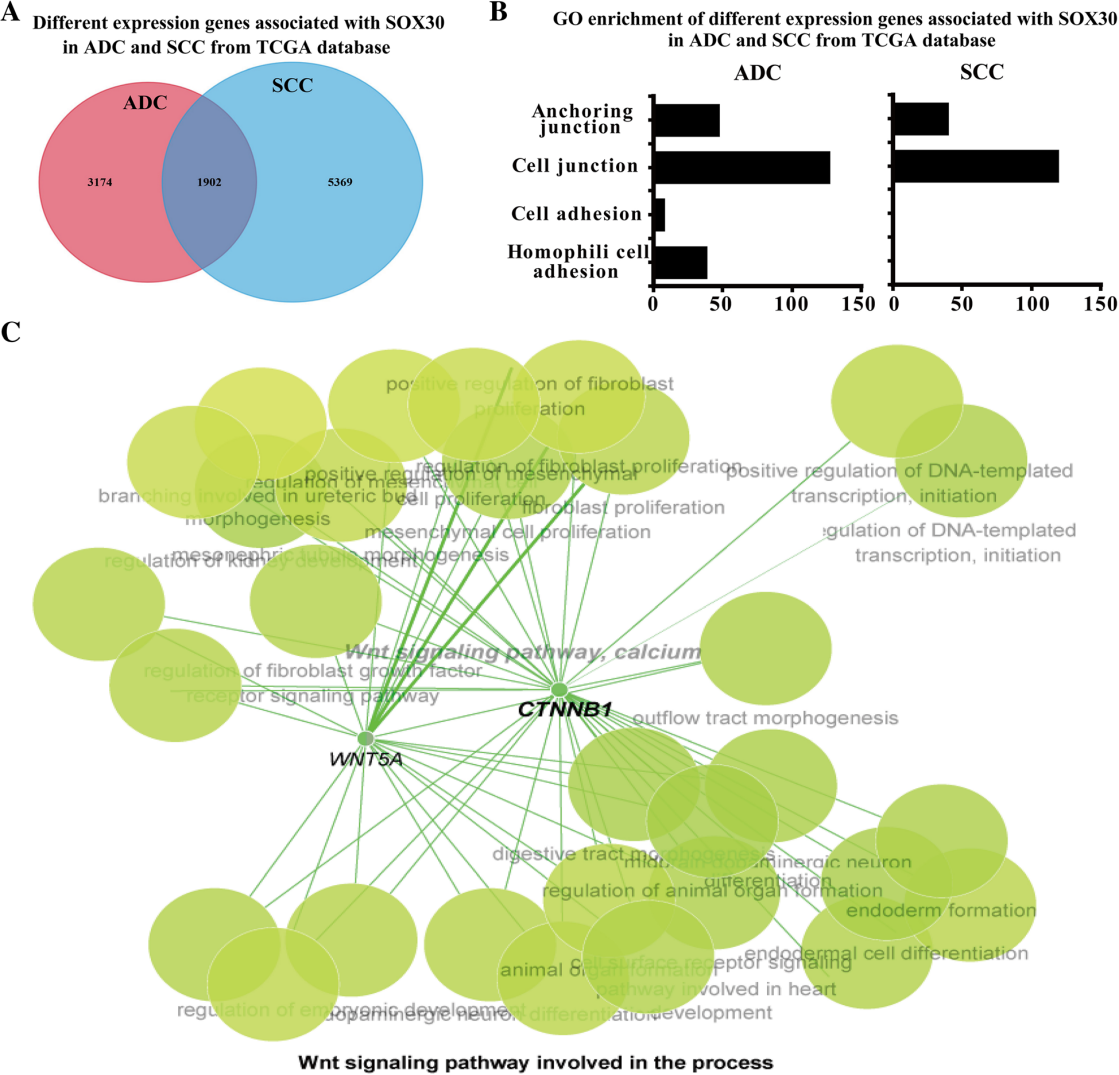
The correlative genes with SOX30 preferentially enrich in metastasis process and Wnt-signaling pathway of ADC patients. **a** The genes significantly associated with SOX30 in ADC and SCC patients from the TCGA database, respectively. Total of 5076 genes and 7271 genes significantly associated with SOX30 was found in ADC and SCC, respectively. **b** GO analyses were performed to enrich different processes of the genes associated with SOX30 in ADC and SCC patients. **c** SOX30-regulated networks were analyzed and the Wnt-signaling was identified as a special module in ADC cell line using targets from RNA array profiles by Cytoscape

To confirm the different roles of SOX30 in ADC and SCC, we downloaded the global expression data of ADC and SCC patients from the TCGA database, and selected the genes significantly associated with SOX30 expression in ADC and SCC patients, respectively. The results indicated that there were 5076 genes and 7271 gene significantly associated with SOX30 expression in ADC and SCC, respectively (*p* < 0.05) (Fig. 3a). In these genes, 1902 genes were shared by ADC and SCC, 3174 genes were only owned by ADC and 5369 genes were only owned by SCC (Fig. 3a). Moreover, GO analyses were employed to determine the genes associated with SOX30 expression involved in functional processes in ADC and SCC, respectively. The prominent difference of the enriched processes in ADC and SCC was the cell adhesion, a key event of tumor metastasis, and these genes preferentially enrich in metastasis process of ADC patients (Fig. 3b).

To determine the possible mechanism for different roles of SOX30 on anti- metastasis in ADC and SCC, we generated global SOX30-regulated networks for potential targets using RNA array profiles in stably transfected ADC (A549) and SCC (H520) cell lines, and identified an important gene network of Wnt/CTNNB1-signaling only in ADC cell line (Fig. 3c). The result reveals that the genes regulated by SOX30 preferentially enrich in Wnt/CTNNB1-signaling pathway in ADC patients.

### SOX30 is negatively associated with CTNNB1 expression in ADC patients not in SCC patients

**Fig. 4:**
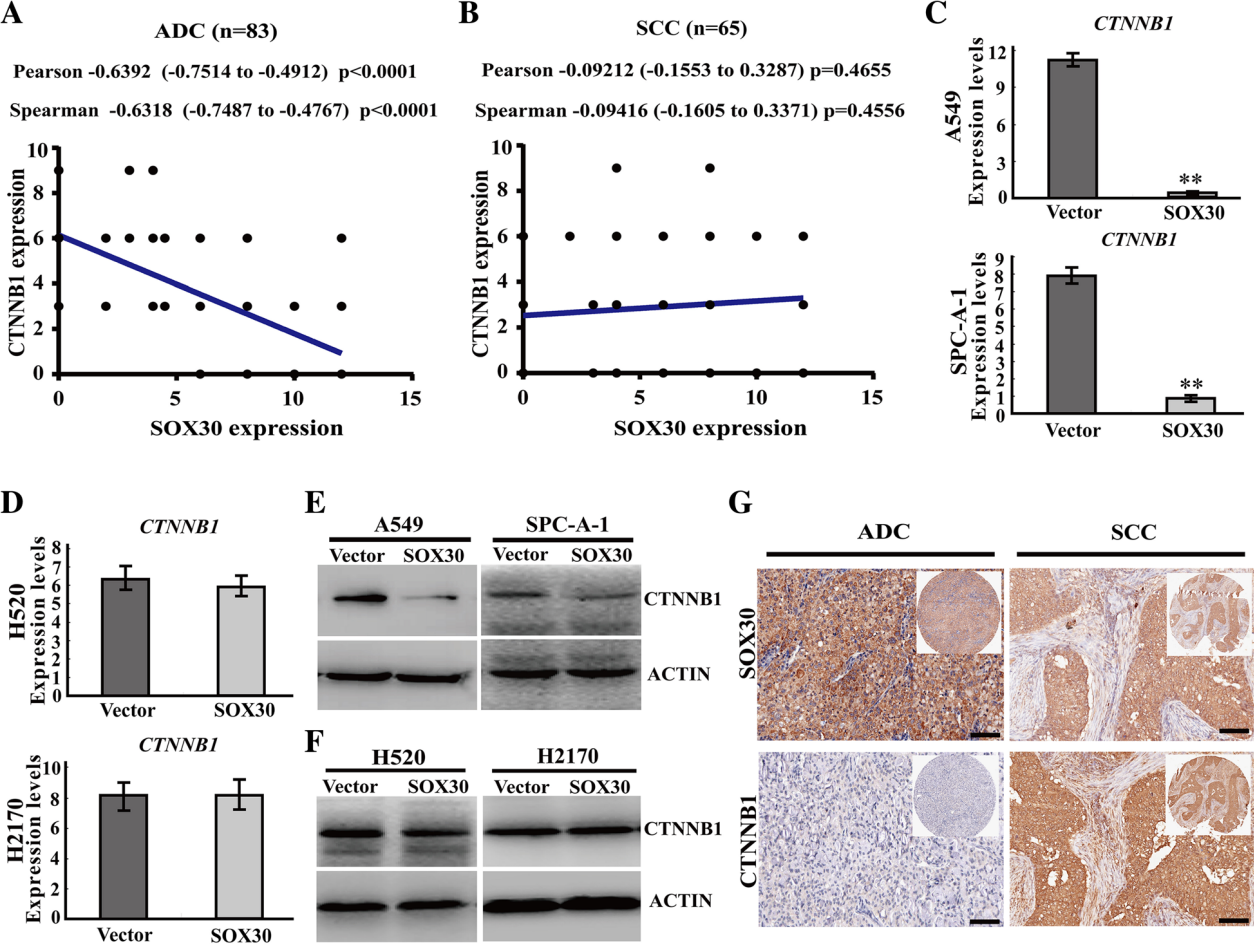
SOX30 is negatively associated with CTNNB1 expression in ADC patients. **a**, **b** SOX30 expression is negatively associated with β-catenin expression in ADC patients but is not associated with β-catenin expression in SCC patients. The *p* value was measured with Pearson and Spearman correlations. **c**-**f** The expression of SOX30 and CTNNB1 was analyzed by RT-qPCR and WB in two ADC cell lines (A549 and SPC-A-1) and two SCC cell lines (H520 and H2170) with or without SOX30. ACTIN was used as an internal control. **g** The relationship of SOX30 and CTNNB1 expression was determined by IHC in ADC and SCC patient’s samples. Scale bars represent 20 μm

To further confirm the possible mechanism for different roles of SOX30 on anti-metastasis in ADC and SCC patients associated with Wnt/CTNNB1-signaling, we determined the relationship between SOX30 expression and CTNNB1 expression in human clinical samples. SOX30 expression was negatively correlated with CTNNB1 (a key metastasis-related gene) expression in ADC patients (*p* < 0.0001) (Fig. 4a), but was not associated with CTNNB1 expression in SCC patients (*p* > 0.05) (Fig. 4b). The percentage of negative CTNNB1 samples in SOX30 high-expression group (60.00%, 18/30) was much higher than in SOX30 low-expression group (22.64%, 12/53) of ADC patients (*p* = 0.001) (Additional file 1: Table S3), however there was no difference of the percentage of negative CTNNB1 between SOX30 high- and low-expression groups of SCC patients (*p* = 0.910) (Additional file 1: Table S3). Moreover, CTNNB1 expression was down-regulated in SOX30-transfected ADC cell lines, whereas it seemed no influence in SOX30-transfected SCC cell lines, H520 and H2170 (Fig. 4d-g) [23]. These results indicate that SOX30 is negatively associated with CTNNB1 expression only in ADC patients.

### SOX30 represses Wnt/CTNNB1-signaling by directly transcriptional inhibition of CTNNB1 in ADC patients

**Fig. 5:**
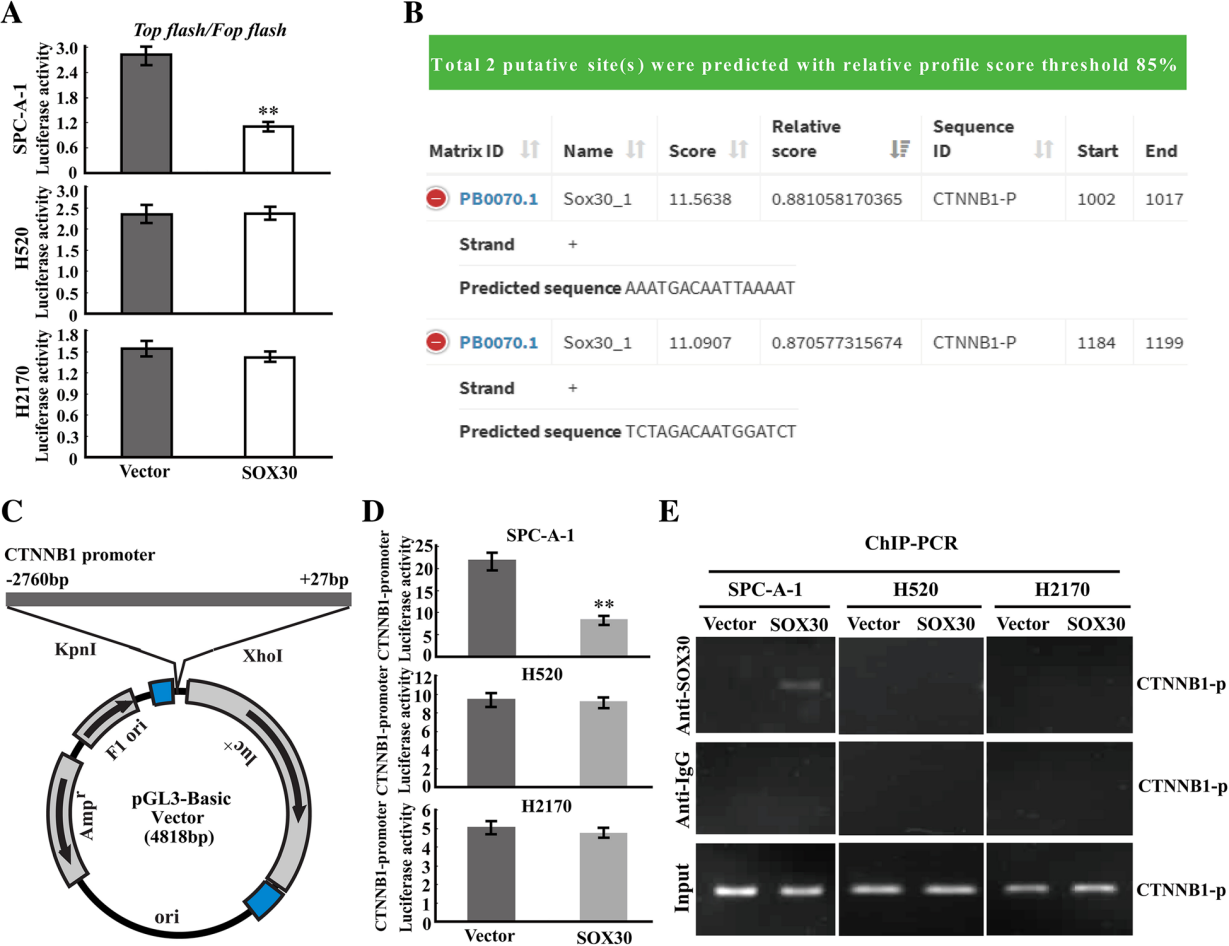
SOX30 suppresses Wnt/CTNNB1 signaling by directly transcriptional inhibition of CTNNB1. **a** SOX30 decreased the TOP-flash reporter activity in SPC-A-1 cells, whereas not affected this activity in H520 and H2170 cells. TOP-flash or FOP-flash plasmid was co-transfected with PRL-TK and SOX30 or empty vector plasmids into the cells, and detected the transcriptional activity of Wnt/CTNNB1 signaling. Error bars indicate SEM. **, *p* < 0.01. The *p* value was obtained by Student’s t-test. **b** Two putative binding sites for SOX30 were predicted in CTNNB1 promoter. It was predicted using Jaspar (http://jaspar.genereg.net/) with the score threshold being 85%. **c** The CTNNB1 promoter was cloned into pGL3-basic luciferase reporter vector. **d** The luciferase reporter assays were performed using the luciferase reporter plamid linked with full-length native promoter of CTNNB1 in SPC-A-1, H520 and and H2170 cells. Error bars indicate SEM. **, *p* < 0.01. The p value was obtained by Student’s t-test. **e** The ChIP-PCR assay was used to determine whether CTNNB1 was a direct target of SOX30 in SPC-A-1, H520 and and H2170 cells

To validate the different relationships between SOX30 and Wnt/CTNNB1-signaling in ADC and SCC patients, we performed TOPflash/FOPflash reporter assays in ADC cell line (SPC-A-1) and SCC cell lines (H520 and H2170). The reporter assays showed that ectopic expression of SOX30 could repress TOPflash/FOPflash transcriptional activity compared with empty vector control in SPC-A-1 cells, but it not changed the activity of TOPflash/FOPflash in H520 and H2170 cells (Fig. 5a). These data reveal that the anti-metastatic role of SOX30 is indeed mediated by inhibiting Wnt/CTNNB1-signaling pathway only in ADC patients.

As SOX30 was a transcriptional factor, we then determined whether SOX30 could regulate CTNNB1 at transcriptional level. Bioinformatic analysis showed that CTNNB1 promoter (− 2760 bp to + 27 bp) contains two potential binding sites (5’-AAATGACAATTAAAAT-3′ and 5’-TCTAGACAATGGATCT-3′) for SOX30 (Fig. 5b), suggesting SOX30 may directly binding to CTNNB1 promoter. We then cloned the CTNNB1 promoter into pGL3-basic luciferase reporter vector (Fig. 5c), and transfected it into SPC-A-1, H520 and H2170 cells with pIRES2-EGFP-SOX30 or pIRES2-EGFP-vector to test the changes of the luciferase activities. The luciferase reporter results revealed that SOX30 significantly attenuated the activity of CTNNB1 promoter in SPC-A-1 cells, but it not changed the activity in H520 and H2170 cells (Fig. 5d), suggesting SOX30 antagonizing Wnt/CTNNB1-signaling by direct binding to CTNNB1 promoter only in ADC. Since there were two potential binding sites for SOX30 in CTNNB1 promoter, we designed two pairs of primers according to the two potential binding sites, and then performed the ChIP-PCR assay. The data of ChIP-PCR assay demonstrated that the direct binding of SOX30 to CTNNB1 promoter of the region including 5’-AAATGACAATTAAAAT-3′ was existed only in ADC cell line, suggesting that the binding sites of 5’-AAATGACAATTAAAAT-3′ in CTNNB1 promoter was required for SOX30 binding (Fig. 5e). These results show that CTNNB1 is a key direct target of SOX30 in ADC patients.

### SOX30 expression has different prognostic values in ADC and SCC patients

**Fig. 6:**
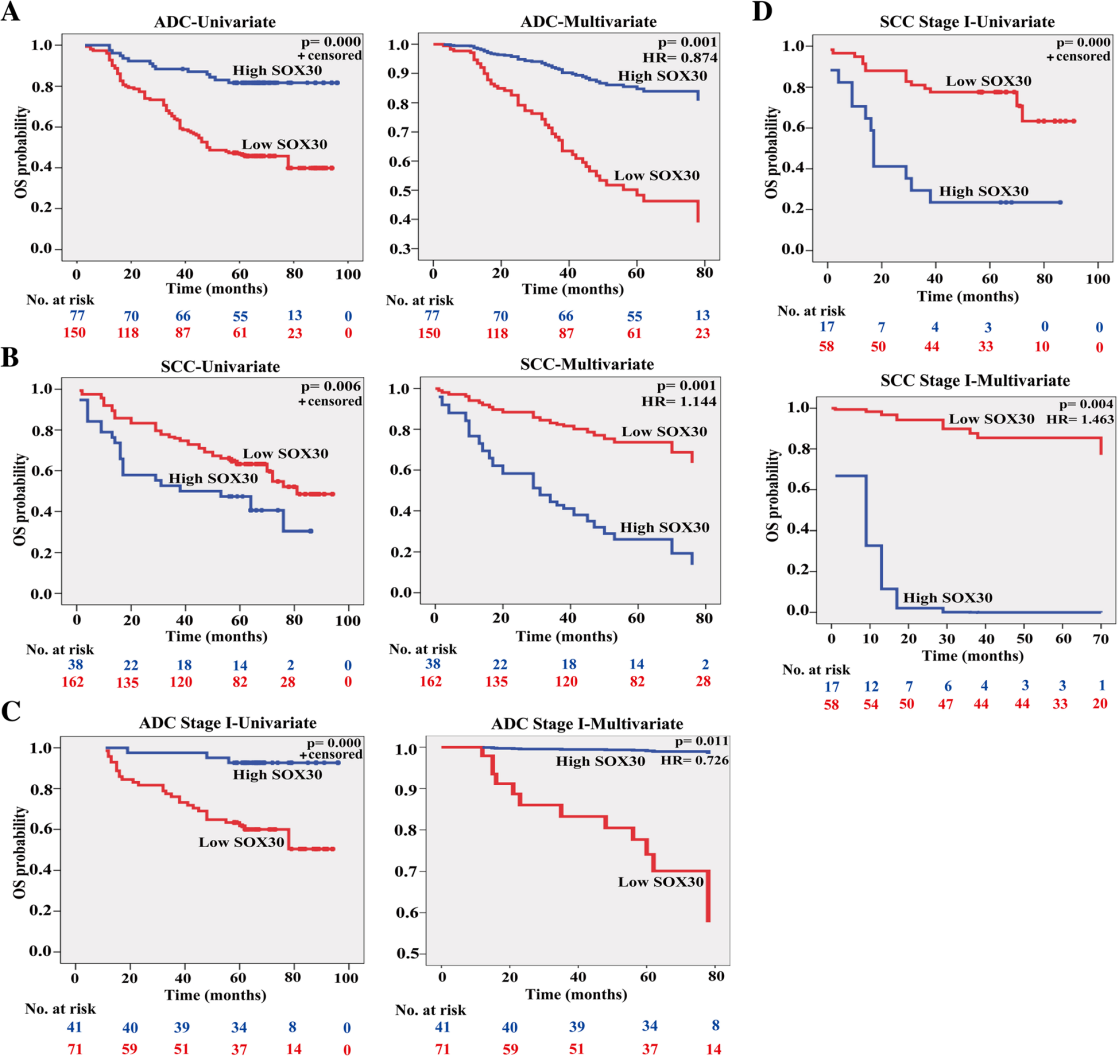
SOX30 is an early-stage prognostic biomarker of ADC and SCC patients. **a** Survival analysis of SOX30 expression in 227 ADC patients split into two groups. Survival analyses were evaluated by Kaplan-Meier survival curve and multivariate Cox regression. HR represents hazard ratio. **b** Survival analysis of SOX30 expression in 200 SCC patients split into two groups. Survival analyses were evaluated by Kaplan-Meier survival curve and multivariate Cox regression. **c** Survival analysis of SOX30 expression in 112 stage I ADC patients split into two groups. Survival analyses were performed using Kaplan-Meier and Cox regression methods. **d** Survival analysis of SOX30 expression in 75 stage I SCC patients split into two groups. Survival analyses were tested by Kaplan-Meier and Cox regression methods

As SOX30 plays different roles on tumor metastasis by inconsistent mechanism in ADC and SCC, we assessed whether the different functions of SOX30 contribute the overall survival (OS) of ADC and SCC patients differently. SOX30 expression was associated with both ADC and SCC patient’s OS by Cox multivariate regression analyses on T/N/M and SOX30 expression, whereas the hazard ratio (HR) was different in ADC (HR < 1.00) and SCC (HR > 1.00) patients (Additional file 1: Table S4), suggesting that SOX30 was a favorable factor for prognosis of ADC patients but was an unfavorable factor for prognosis of SCC patients. To confirm these results, we used a Kaplan-Meier log rank test analysis to reveal a poor OS in ADC patients characterized with SOX30-low expression (*p* = 0.000), but a good OS in SCC patients characterized with SOX30-low expression (*p* = 0.006) (Fig. 6a and b, Additional file 2: Figure S1A and B). To correct for bias, SOX30 expression as well as other parameters, such as age, clinical stage, gender, histological grade, tumor diameter, tumor location and lymph node, were examined in a multivariate Cox-regression analysis. SOX30 expression was a favorable and independent prognostic factor for OS of ADC patients (HR = 0.874, *p* = 0.001), but was an unfavorable and independent prognostic factor for OS of SCC patients (HR = 1.144, *p* = 0.001) (Fig. 6a and b, Table 2, Additional file 2: Figure S1A and B, Additional file 1: Table S5). These data reveal that different metastatic roles of SOX30 have different prognostic values in ADC and SCC patients.

**Table 2:** Multivariate analysis of different prognostic factors in all stages and stage I ADC patients (two groups)

ADC type	Variable	Comparison	Hazard ratio (95% CI)	*P* value
All stages ADC patients (*n* = 275)	Age	20–84 years	1.057 (1.025–1.090)	**0.000**
	Gender	Male; Female	1.581 (0.789–3.169)	0.196
	Histological grade	Grade 1–3	1.107 (0.712–1.722)	0.652
	Tumor diameter	0.8–8 cm	0.973 (0.788–1.201)	0.796
	Lymph node no	0–15	1. 014 (0.939–1.096)	0.720
	Clinical stage	Stage I- IV	2.605 (1.852–3.663)	**0.000**
	Tumor location	Left; Right	0.535 (0.298–0.960)	**0.036**
	SOX30 expression	High; Low	0.874 (0.807–0.946)	**0.001**
Stage I ADC patients (*n* = 112)	Age	20–84 years	1.109 (1.011–1.216)	**0.028**
	Gender	Male; Female	1.182 (0.155–9.002)	0.872
	Histological grade	Grade 1; 2; 3	4. 702 (0.586–37.737)	0.145
	Tumor diameter	0.8-7 cm	0.954 (0.402–2.268)	0.916
	Tumor location	Left; Right	2. 605 (0.642–10.572)	0.180
	SOX30 expression	High; Low	0.726 (0.567–0.929)	**0.011**

Cox regression analysis was used to test independent prognostic contributions

CI represents confidence interval. The *p* < 0.05 was considered statistically significant

The entries in boldface indicate statistically significant

### SOX30 represents an early-stage favorable prognostic biomarker of ADC patients

Then the correlations between SOX30 expression and OS of ADC or SCC patients at different clinical stages were evaluated. SOX30 expression was obviously associated with the OS of ADC patients at clinical stage I using both Kaplan-Meier (*p* = 0.000) and Cox-Regression (HR = 0.726, *p* = 0.011) analyses (Fig. 6c, Table 2, Additional file 2: Figure S2A). SOX30 expression was associated with the OS of ADC patients at clinical stage II only using Kaplan-Meier analysis (*p* = 0.029) but not using Cox-Regression analysis (*p* = 0.668). SOX30 expression was not associated with OS of the ADC patients at clinical stage III + IV (*p* = 0.152). Strikingly, SOX30 expression was also obviously associated with OS of the SCC patients at clinical stage I using both Kaplan-Meier (*p* = 0.000) and Cox-Regression (HR = 1.463, *p* = 0.004) analyses (Fig. 6d, Additional file 2: Figure S2B, Additional file 1: Table S5), but not of the SCC patients at clinical stage II (*p* = 0.567) or III + IV (*p* = 0.563). SOX30 represented a favorable and independent prognostic biomarker of the stage I ADC patients, but represented an unfavorable and independent prognostic biomarker of the stage I SCC patients (Fig. 6c and d, Table 2, Additional file 2: Figure S2A and B, Additional file 1: Table S5). These data indicate that SOX30 is a double face early-stage prognostic biomarker, a favorable prognostic marker of the ADC patients at stage I and an unfavorable prognostic marker of the SCC patients at stage I.

### Forcible restoration of CTNNB1 inhibits the anti-metastatic role of SOX30

**Fig. 7:**
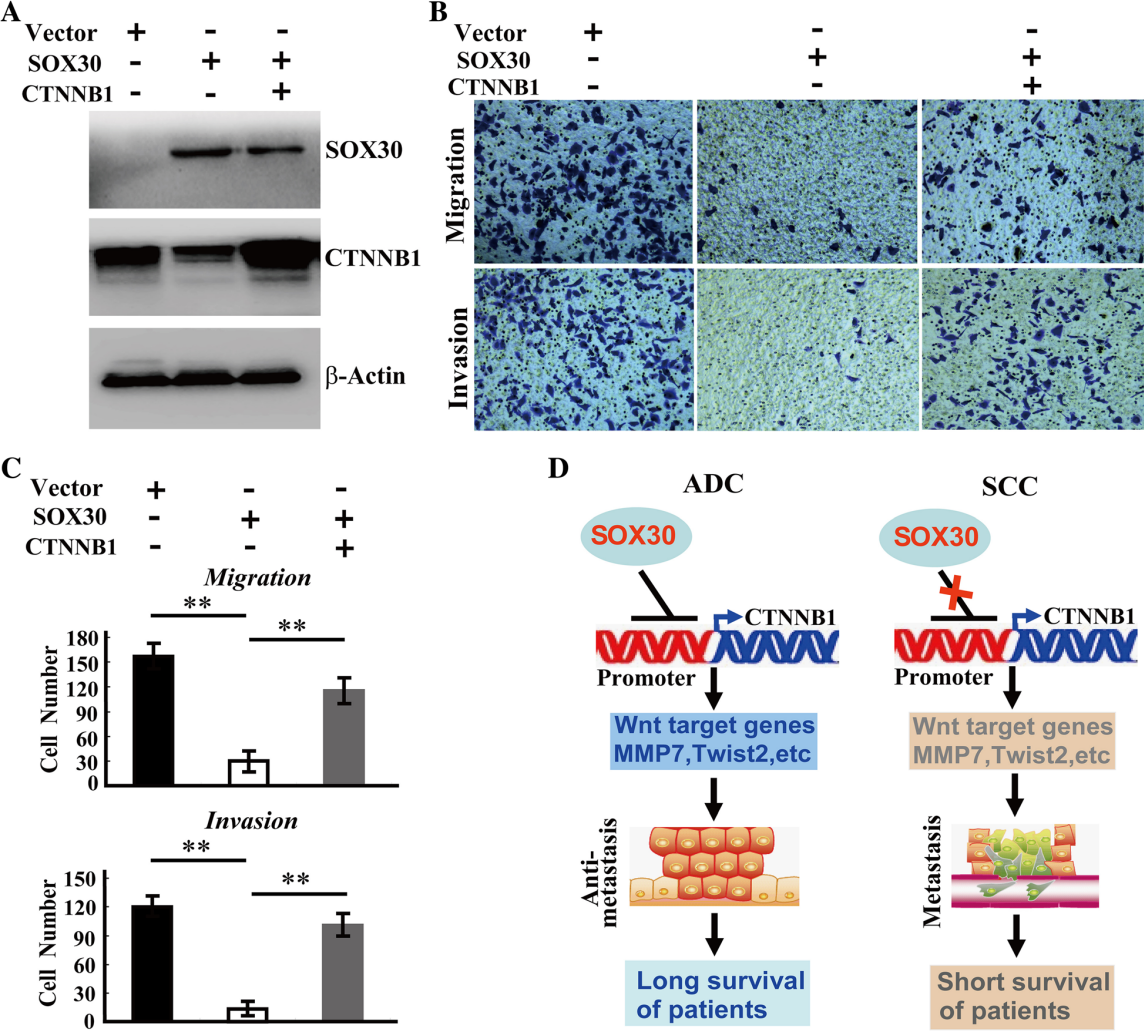
CTNNB1 is an important target in the anti-metastatic function of SOX30 in ADC patients. **a** SOX30 and CTNNB1 expressions were analyzed at 48 h after transfected with empty-vector, SOX30 and co-transfected with SOX30 and CTNNB1 plasmids in SPC-A-1 cells by WB. **b**, **c** The cell migration and invasion were determined at 48 h after transfected with empty-vector, SOX30 and co-transfected with SOX30 and CTNNB1 plasmids in SPC-A-1 cells by transwell assays. The numbers of migrated or invaded cells were quantified by average count of five random microscopic fields. **, *p* < 0.01. The p value was obtained by Student’s t-tests. **d** The mechanism illustration for different roles on tumor metastasis of SOX30 in ADC and SCC patients

To determine the importance of CTNNB1 in the anti-metastatic function of SOX30 in ADC, we enforced CTNNB1 expression when over-expression of SOX30 in SPC-A-1 cells (Fig. 7a), and detected the restoration of SPC-A-1 cell migration and invasion. Forcible restoration of CTNNB1 when over-expression of SOX30 could greatly diminish the anti-metastatic role of SOX30 in SPC-A-1 cells (Fig. 7b and c). These results reveal that CTNNB1 is an important target for SOX30 acting as a tumor metastasis suppressor.

## Discussion

The high-mortality of NSCLC patients is greatly due to distant metastasis [28, 29]. Different subtypes of NSCLC usually show different molecular events of metastases and different responses of therapeutic strategy, which highlights the importance and urgency of characterizing molecular abnormalities in different tumor-types to search for novel special therapeutic targets and prolong the survival of cancer patients [10–14]. In the present study, clinical analyses reveal that SOX30 is significantly associated with metastasis of ADC patients, but not associated with metastasis of SCC patients. Functional analyses indicate that SOX30 strongly inhibits tumor cell metastasis in ADC cell lines, but not affects tumor cell metastasis in SCC cell lines, which is consistent with different association of SOX30 with metastasis in ADC and SCC. Mechanistically, SOX30 is negatively associated with β-catenin expression, a key metastatic gene, only in ADC, and SOX30 indeed inhibits Wnt/CTNNB1-signaling pathway by direct binding to CTNNB1 promoter only in ADC. These data indicate that SOX30 plays a specific role on tumor metastasis via directly transcriptional repression of CTNNB1 in ADC patients.

In the clinical, more than 60% of NSCLC patients are diagnosed at advanced stage. The five-year survival rate of the patients at advanced stage is less than 10%, whereas it is greater than 70% of the patients at clinical stage I [30, 31], indicating that identification of prognostic biomarkers for early-stage is of great importance to prolong NSCLC patient’s survival. In our present study, Kaplan-Meier and Cox regression analyses revealed that SOX30 expression was significantly correlated with ADC patient’s survival, especially with OS of the ADC patients at stage I. The elevated SOX30 expression inhibited tumor-metastasis and represented as a favorable prognostic biomarker of the ADC patients at stage I. These data suggest that SOX30 serves as a new favorable early-stage prognostic candidate for ADC patients, which can be used to accurately predict clinical outcomes of ADC patients.

In our previous study, we have also found that SOX30 expression is correlated with histological types of NSCLC, and is a favorable prognostic factor in ADC patients (*n* = 150) but not in SCC patients (*n* = 70) [20]. In the present study, we further corroborated these results in a new larger cohort (*n* = 275 for ADC and *n* = 231 for SCC). In previous study, a better OS for the ADC patients with SOX30-high expression was seen only in the subset of stage II not in stage I [20], which seemed to be very confusing and might be due to the very small sample size. In the new cohort with larger sample size of our present study, SOX30 expression is also associated with the OS of ADC patients at clinical stage II using univariate analysis (*p* = 0.029) but not using multivariate analysis (*p* = 0.668). Most importantly, the difference in our present study from previous study is that SOX30 expression is obviously associated with the OS of ADC patients at clinical stage I (Kaplan-Meier analysis, *p* = 0.000; Cox-Regression analysis, *p* = 0.011). These data reveal that SOX30 is an early-stage favorable prognostic biomarker for ADC patients in fact.

To ensure SOX30 is indeed an early-stage prognostic marker for ADC patients, we further determined the reliability of the result that SOX30 is a favorable early stage prognostic factor for ADC carefully. As SOX30 was poorly expressed in the patients at late stage and the expression was categorized as high or low according to the median score of the patients at all clinical stages, the number of SOX30-high expression patients at advanced stage is only 12, which is really small. Unfortunately, it is difficult to increase the number of the patients at late stage in a short time due to very rare of patients underwent surgery when diagnosed as advanced stage. As a complement, we reanalyzed the prognostic value of SOX30 in the patients at late stage after categorizing the patients as SOX30-high or -low group according to the median score of the patients at only stage III + IV (the number of high SOX30 expression patients at late stage is 40, and if SOX30 expression is associated with the survival of ADC patients at advanced stage, it should also be reflected in the regrouped patients base on the median score of only late stage patients). The result showed that SOX30 expression is still not associated with survival of the ADC patients at late stage (*p* = 0.718). Thus, SOX30 is indeed a favorable prognostic factor for ADC patients at early stage, but not for the patients at advanced stage. SOX30 as a tumor metastasis suppressor is a favorable prognostic marker for the ADC patients at early stage, indicating that SOX30 has acted important role in the tumor onset of early stage, which further shows the powerful role of SOX30 in ADC.

SOX30 has different prognostic values in ADC and SCC patients at early stage. From the data of our present study, the reason is that SOX30 plays different roles on tumor metastasis as differently direct regulation of CTNNB1. In early-stage ADC patients, the elevated SOX30 represses Wnt/CTNNB1-signaling by directly binding to CTNNB1 promoter to inhibit tumor-metastasis and results in a favorable prognosis of these patients. While in early-stage SCC patients, SOX30 has no role on tumor-metastasis and Wnt/CTNNB1-signaling duo to not binding to CTNNB1 promoter and results in an unfavorable prognosis of the patients. However, why SOX30 can not bind to CTNNB1 promoter in SCC patients is still unknown. Is there any mutation/deletion in the key binding sites of SOX30 binding to CTNNB1 promoter or whether the key binding sites of SOX30 binding to CTNNB1 promoter was occupied by other factors in SCC patient?., Further studies are required to clarify this interesting issue. Another remaining question is that SOX30 do not affect the tumor metastasis in SCC, how it can become an unfavorable prognostic factor for OS of SCC patients? Further studies are required to explain this problem.

## Conclusions

SOX30 is a key metastatic suppressor with inhibition of Wnt/CTNNB1-signaling by directly binding to CTNNB1 promoter to prolong the survival of ADC patients, whereas SOX30 plays no inhibitory role on tumor-metastasis without suppression of Wnt/CTNNB1-signaling duo to not binding to CTNNB1 promoter resulting in short survival of SCC patients (Fig. 7d). The novelly specific role of SOX30 in ADC provides specifically effective therapies against tumor metastasis and useful information for prolonging ADC patient’s survival.

## Additional files

Additional file 1: **Table S1.** Correlation of SOX30 expression with clinicopathologic features in NSCLC patients (*n* = 537). **Table S2.** Correlation of SOX30 expression with clinicopathologic features in SCC patients (*n* = 228). **Table S3.** Correlation of SOX30 with CTNNB1 expression in ADC and SCC patients (*n* = 148). **Table S4.** Multivariate analyses of TNM prognostic factors and SOX30 expression for overall survival (OS) of 275 ADC and 228 SCC patients. **Table S5.** Multivariate analysis of different prognostic factors in all stages and stage I SCC patients (two groups). (DOC 77 kb)

Additional file 2: **Figure S1.** SOX30 expression has different prognostic values in ADC and SCC. (A) Survival analysis of SOX30 expression in 227 ADC patients split into three groups. Survival analyses were evaluated by Kaplan-Meier survival curve and multivariate Cox regression. HR represents hazard ratio. SOX30 high, scores 8 < and ≤ 12; SOX30 medium, score 8; SOX30 low, scores < 8. (B) Survival analysis of SOX30 expression in 200 SCC patients split into three groups. Survival analyses were evaluated by Kaplan-Meier survival curve and multivariate Cox regression. **Figure S2.** SOX30 is an early-stage prognostic biomarker for ADC or SCC patients. (A) Survival analysis of SOX30 expression in 112 stage I ADC patients split into three groups. Survival analyses were performed using Kaplan-Meier and Cox regression methods. (B) Survival analysis of SOX30 expression in 75 stage I SCC patients split into three groups. Survival analyses were tested by Kaplan-Meier and Cox regression methods. (DOC 4958 kb)

## Abbreviations

ADC: Adenocarcinoma
ChIP: Chromatin- immunoprecipitation
CTNNB1: β-catenin
GO: Gene ontology
HR: Hazard ratio
IHC: Immunohistochemistry
LCC: Large cell carcinoma
NSCLC: Non-small cell lung cancer
OS: Overall survival
SCC: Squamous cell carcinoma
WB: Western blotting
