# Supplementary material for: SOX30 specially prevents Wnt-signaling to suppress metastasis and improve prognosis of lung adenocarcinoma patients
Source: Respir Res. 2018 Dec 4;19:241. doi: 10.1186/s12931-018-0952-3 (PMC6280504; doi:10.1186/s12931-018-0952-3)
Supplement: Supplementary file 1 — Table S1. Correlation of SOX30 expression with clinicopathologic features in NSCLC patients (n = 537). Table S2. Correlation of SOX30 expression with clinicopathologic features in SCC patients (n = 228). Table S3. Correlation of SOX30 with CTNNB1 expression in ADC and SCC patients (n = 148). Table S4. Multivariate analyses of TNM prognostic factors and SOX30 expression for overall survival (OS) of 275 ADC and 228 SCC patients. Table S5. Multivariate analysis of different prognostic factors in all stages and stage I SCC patients (two groups). (DOC 77 kb) [file 12931_2018_952_MOESM1_ESM.doc]

**Additional file 1**

**Table S1 Correlation of SOX30 expression with clinicopathologic features in NSCLC patients (n=537**)

| SOX30 Expression  Clinical Feature Total (no.) High (n=158) Low (n=379) P value | | | | |
| --- | --- | --- | --- | --- |
| Histological type  LCC  ADC  SCC | 34  275  228 | 21  94  43 | 13  181  185 | **0.000** |

The p value was measured with Pearson chi-square tests. The statistical tests are two sided.

**Table S2 Correlation of SOX30 expression with clinicopathologic features in SCC patients** (n=228)

| SOX30 Expression  Clinical Feature Total High (n=43) Low (n=185) P value | | | | |
| --- | --- | --- | --- | --- |
| Age (years)  ≤60  >60 | 87  139 | 18  25 | 69  114 | 0.614 |
| Clinical stage  I  II  III-IV | 77  60  50 | 17  13  6 | 60  47  44 | 0.315 |
| Tumor size  T1-2  T3-4 | 155  52 | 33  5 | 122  47 | 0.060 |
| Lymph node status  N0  N1-3 | 104  83 | 22  14 | 82  69 | 0.460 |
| Metastasis  M0  M1 | 205  2 | 38  0 | 167  2 | 0.500 |
| Gender  Male  Female | 212  16 | 37  5 | 175  11 | 0.170 |
| Histological grade  1  2  3 | 25  114  86 | 1  15  26 | 24  99  60 | **0.001** |
| Tumor diameter  ≤ 4cm  > 4cm | 118  97 | 18  22 | 100  75 | 0.164 |
| Location  Left  Right | 97  131 | 15  28 | 82  103 | 0.259 |

The p values were measured with Pearson chi-square tests.

The tumor clinical stage, tumor status, lymph node status and metastasis were classified according to the international system.

All statistical tests are two sided

**Table S3 Correlation of SOX30 with CTNNB1 expression in ADC and SCC patients (n=148**)

| SOX30 Expression  Clinical Feature Total (no.) High (n=48) Low (n=100) P value | | | | |
| --- | --- | --- | --- | --- |
| ADC CTNNB1  Negative  Positive | 30  53 | 18  12 | 12  41 | **0.001** |
| SCC CTNNB1  Negative  Positive | 26  39 | 7  11 | 19  28 | 0.910 |

The p values were measured with Pearson chi-square tests.

All statistical tests are two sided.

**Table S4 Multivariate analyses of TNM prognostic factors and SOX30 expression for overall survival (OS) of 275 ADC and 228 SCC patients**

| Histology | Variables | Comparison | Hazard Ratio (95%CI) | p-value |
| --- | --- | --- | --- | --- |
| ADC | T | T1-2; T3-4 | 1.133 (0.854-1.503) | 0.386 |
|  | N | N0; N1-3 | 2.305 (1.485-3.580) | **0.000** |
|  | M | M0; M1 | 1.772(0.695-4.516) | 0.231 |
|  | SOX30 | High; Low | 0.883 (0.833-0.935) | **0.000** |
| SCC | T | T1-2; T3-4 | 1.205 (0.948-1.533) | 0.127 |
|  | N | N0; N1-3 | 1.050 (0.677-1.630) | 0.827 |
|  | M | M0; M1 | 4.239 (0.960-18.724) | 0.057 |
|  | SOX30 | High; Low | 1.085 (1.037-1.134) | **0.000** |

Cox regression analysis was used to test independent prognostic contribution of SOX30 after accounting for other potentially important covariates.

CI represents confidence interval. The p<0.05 was considered statistically significant.

**Table S5 Multivariate analysis of different prognostic factors in all stages and stage I SCC patients (two groups)**

| SCC type | Variable | Comparison | Hazard Ratio (95%CI) | P value |
| --- | --- | --- | --- | --- |
| All stages SCC patients (n=228) | Age | 33-82 years | 1.090 (1.035-1.148) | **0.001** |
|  | Gender | Male; Female | 2.214 (0.800-6.128) | 0.126 |
|  | Histological grade | Grade 1-3 | 1. 077 (0.634-1.829) | 0.785 |
|  | Tumor diameter | 0.5-13cm | 0.932 (0.763-1.138) | 0.488 |
|  | Lymph node no | 0-20 | 0. 855 (0.644-1.136) | 0.280 |
|  | Clinical stage | Stage I-IV | 2.769 (1.345-5.697) | **0.006** |
|  | Tumor location | Left; Right | 2.007 (0.999-4.031) | **0.050** |
|  | SOX30 expression | High; Low | 1.144 (1.055-1.240) | **0.001** |
| Stage I SCC patients (n=75) | Age | 33-82 years | 1.020 (0.953-1.091) | 0.566 |
|  | Gender | Male; Female | 0.465(0.068-3.199) | 0.436 |
|  | Histological grade | Grade 1; 2; 3 | 1. 199 (0.294-4.882) | 0.800 |
|  | Tumor diameter | 0.5-8cm | 1.039 (0.434-2.486) | 0.932 |
|  | Tumor location | Left; Right | 2.124 (0.428-10.533) | 0.356 |
|  | SOX30 expression | High; Low | 1.463 (1.132-1.890) | **0.004** |
