# Supplementary material for: SOX30 specially prevents Wnt-signaling to suppress metastasis and improve prognosis of lung adenocarcinoma patients
Source: Respir Res. 2018 Dec 4;19:241. doi: 10.1186/s12931-018-0952-3 (PMC6280504; doi:10.1186/s12931-018-0952-3)
Supplement: Supplementary file 2 — Figure S1. SOX30 expression has different prognostic values in ADC and SCC. (A) Survival analysis of SOX30 expression in 227 ADC patients split into three groups. Survival analyses were evaluated by Kaplan-Meier survival curve and multivariate Cox regression. HR represents hazard ratio. SOX30 high, scores 8 < and ≤ 12; SOX30 medium, score 8; SOX30 low, scores < 8. (B) Survival analysis of SOX30 expression in 200 SCC patients split into three groups. Survival analyses were evaluated by Kaplan-Meier survival curve and multivariate Cox regression. Figure S2. SOX30 is an early-stage prognostic biomarker for ADC or SCC patients. (A) Survival analysis of SOX30 expression in 112 stage I ADC patients split into three groups. Survival analyses were performed using Kaplan-Meier and Cox regression methods. (B) Survival analysis of SOX30 expression in 75 stage I SCC patients split into three groups. Survival analyses were tested by Kaplan-Meier and Cox regression methods. (DOC 4958 kb) [file 12931_2018_952_MOESM2_ESM.doc]

**Additional file 2**


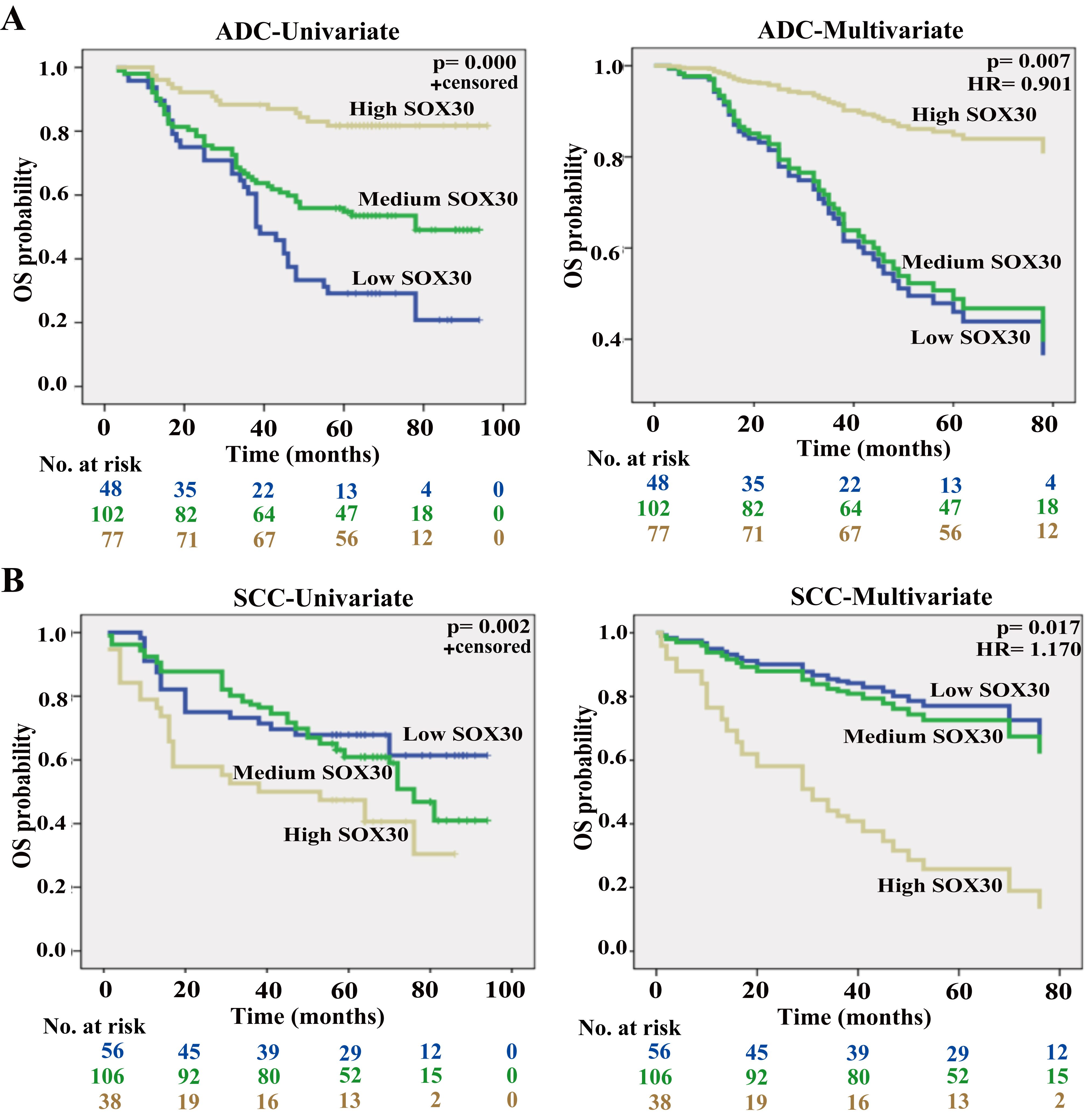


**Figure S1 SOX30 expression has different prognostic values in ADC and SCC**

(A) Survival analysis of SOX30 expression in 227 ADC patients split into three groups. Survival analyses were evaluated by Kaplan-Meier survival curve and multivariate Cox regression. HR represents hazard ratio. SOX30 high, scores 8< and ≤12; SOX30 medium, score 8; SOX30 low, scores <8. (B) Survival analysis of SOX30 expression in 200 SCC patients split into three groups. Survival analyses were evaluated by Kaplan-Meier survival curve and multivariate Cox regression.

**
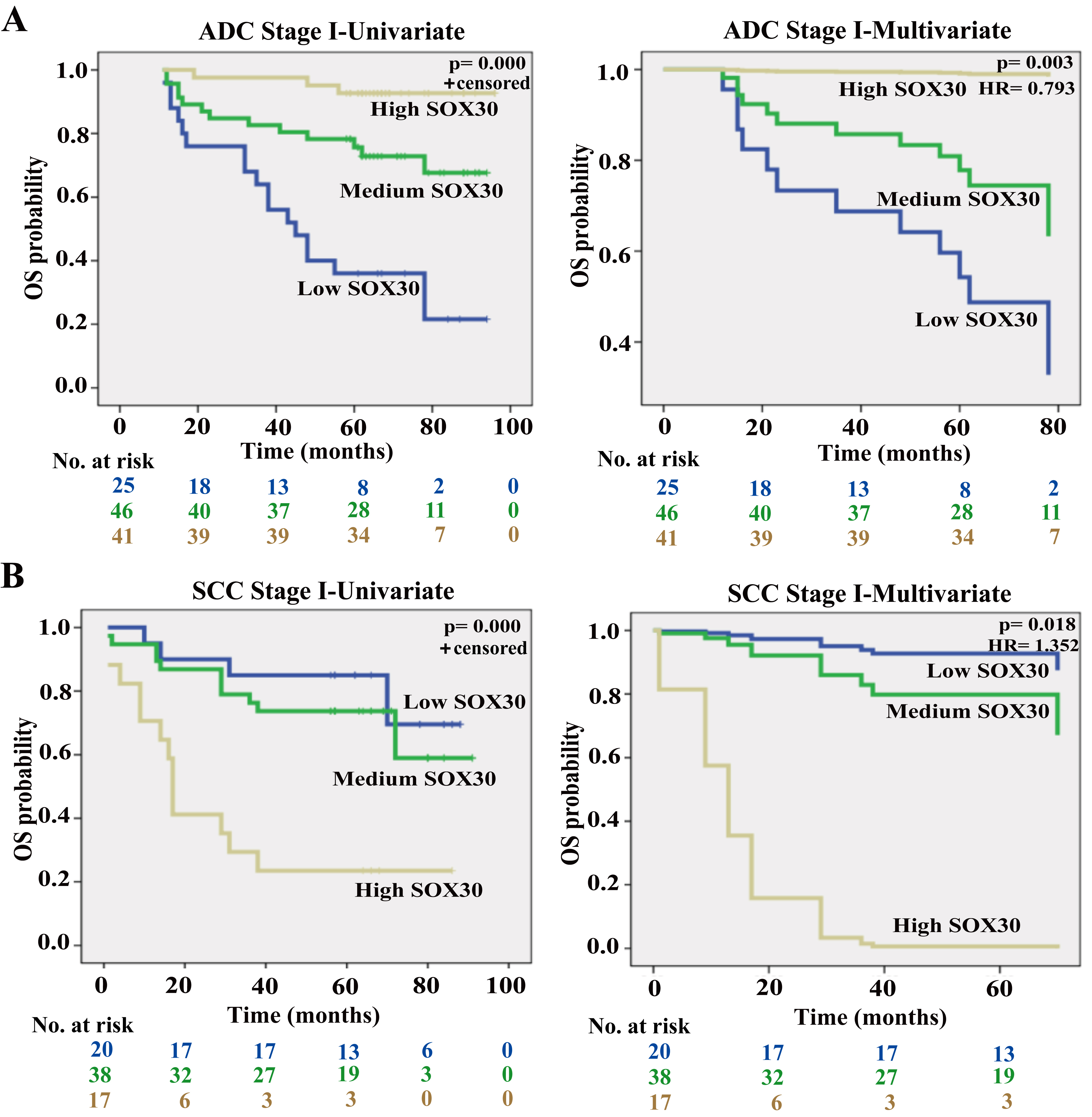
**

**Figure S2 SOX30 is an early-stage prognostic biomarker for ADC or SCC patients**

(A) Survival analysis of SOX30 expression in 112 stage I ADC patients split into three groups. Survival analyses were performed using Kaplan-Meier and Cox regression methods. (B) Survival analysis of SOX30 expression in 75 stage I SCC patients split into three groups. Survival analyses were tested by Kaplan-Meier and Cox regression methods.
